# Large-scale deletions of the *ABCA1* gene in patients with hypoalphalipoproteinemia[S]

**DOI:** 10.1194/jlr.P086280

**Published:** 2018-06-04

**Authors:** Jacqueline S. Dron, Jian Wang, Amanda J. Berberich, Michael A. Iacocca, Henian Cao, Ping Yang, Joan Knoll, Karine Tremblay, Diane Brisson, Christian Netzer, Ioanna Gouni-Berthold, Daniel Gaudet, Robert A. Hegele

**Affiliations:** Robarts Research Institute,* Schulich School of Medicine and Dentistry, Western University, London ON, Canada; Department of Biochemistry,† Schulich School of Medicine and Dentistry, Western University, London ON, Canada; Department of Medicine,§ Schulich School of Medicine and Dentistry, Western University, London ON, Canada; Department of Pathology and Laboratory Medicine,** Schulich School of Medicine and Dentistry, Western University, London ON, Canada; ^5^ Lipidology Unit,†† Community Genomic Medicine Centre and ECOGENE-21, Department of Medicine, Université de Montréal, Saguenay QC, Canada; Institute for Human Genetics,§§ University of Cologne, Germany; Polyclinic for Endocrinology, Diabetes and Preventive Medicine,*** University of Cologne, Germany

**Keywords:** ATP-binding cassette subfamily A member 1, bioinformatic analysis, copy-number variation, high density lipoprotein cholesterol, next-generation sequencing, diagnostic tools, genetic testing, dyslipidemia

## Abstract

Copy-number variations (CNVs) have been studied in the context of familial hypercholesterolemia but have not yet been evaluated in patients with extreme levels of HDL cholesterol. We evaluated targeted, next-generation sequencing data from patients with very low levels of HDL cholesterol (i.e., hypoalphalipoproteinemia) with the VarSeq-CNV^®^ caller algorithm to screen for CNVs that disrupted the *ABCA1*, *LCAT*, or *APOA1* genes. In four individuals, we found three unique deletions in *ABCA1*: a heterozygous deletion of exon 4, a heterozygous deletion that spanned exons 8 to 31, and a heterozygous deletion of the entire *ABCA1* gene. Breakpoints were identified with Sanger sequencing, and the full-gene deletion was confirmed by using exome sequencing and the Affymetrix CytoScan HD array. Previously, large-scale deletions in candidate HDL genes had not been associated with hypoalphalipoproteinemia; our findings indicate that CNVs in *ABCA1* may be a previously unappreciated genetic determinant of low levels of HDL cholesterol. By coupling bioinformatic analyses with next-generation sequencing data, we can successfully assess the spectrum of genetic determinants of many dyslipidemias, including hypoalphalipoproteinemia.

Extremely low levels of HDL cholesterol, clinically characterized as “hypoalphalipoproteinemia”, can result from various molecular etiologies. DNA sequencing of candidate genes has shown that between ∼10–35% of affected individuals have rare heterozygous missense, nonsense, or splicing variants in *ABCA1*, *APOA1*, and *LCAT* genes, encoding ABCA1, apo A-I, and lecithin:cholesterol acyl transferase, respectively (1–6). We recently found that another ∼18% of affected individuals have an extreme polygenic accumulation of common variants, as quantified by a polygenic trait score that considers several common SNPs associated with HDL cholesterol levels (1). However, the genetic basis of low HDL cholesterol in the majority of individuals with hypoalphalipoproteinemia remains to be characterized.

Copy-number variations (CNVs) are deletions and duplications of genomic material that are much larger than single nucleotide variations (SNVs); by convention, “CNVs” are deletions or duplications >50 bp in size (7). While CNVs have been commonly identified throughout the genome, there has been a surging focus on CNVs that are rare within the population, and their relationship to certain phenotypes and diseases (8). This redefined focus has been due to improvements in bioinformatic tools and targeted next-generation sequencing (NGS) panels designed for clinical utility. Previously, specialized molecular methods, such as multiplex ligation-dependent probe amplification (MLPA), have been required to detect CNVs, and had to be performed concurrently with other genetic methods. Now, through the development of new bioinformatic methods, CNVs can be easily screened for in patient groups using data generated by a single genetic approach; namely, NGS. We recently reported that data generated by a targeted NGS panel designed to detect SNVs in genes related to familial hypercholesterolemia (FH) could be processed with dedicated bioinformatic tools to diagnose the presence of CNVs in *LDLR*, encoding the LDL receptor. Results of our NGS-based CNV detection method showed 100% concordance with traditional MLPA of *LDLR*, with no false negative or false positive results (9).

CNVs disrupting *ABCA1*, *APOA1*, or *LCAT* in individuals with hypoalphalipoproteinemia have not yet been reported. Here, we applied our novel bioinformatic approach to previously generated targeted NGS data from patients with hypoalphalipoproteinemia, with particular interest in patients without rare variants in HDL-associated genes or without an extreme polygenic accumulation of common variants (10). Out of 288 patients screened, we found four patients who had one of three novel heterozygous CNVs within the *ABCA1* gene; the variants were confirmed using independent methods. Our findings not only demonstrate the usefulness of applying bioinformatically-based CNV calling algorithms to NGS data, but we also provide the first example of large-scale CNV deletions that are likely causing hypoalphalipoproteinemia.

## METHODS

### Study subjects

Patients who were referred to the Lipid Genetics Clinic at the London Health Sciences Centre, University Hospital (London ON, Canada) for “low HDL cholesterol” or “hypoalphalipoproteinemia” were considered for this screening study. Patients provided signed consent with approval from the Western University ethics review board (no. 07290E).

### Targeted next-generation sequencing

Genomic DNA isolation, sample preparation, and targeted sequencing using our “LipidSeq” panel have been described in detail previously (1, 11).

### Bioinformatic processing of sequencing data

DNA sequence data in the form of FASTQ files were imported into CLC Bio Genomics Workbench (version 8.5; CLC Bio, Aarhus, Denmark) for bioinformatic processing. The sequencing data was aligned to the human reference genome (build hg19), depth of coverage was exported as a BAM file, and any identified variants were exported to VCF files for each patient.

### Identification of copy-number and single nucleotide variations

The BAM and VCF files generated for each patient were imported into VarSeq® (version 1.4.8; Golden Helix, Inc., Bozeman, MT) for annotation of each genetic variant. SNVs were identified following methods that have been described in detail previously (1). Assessment of CNVs in *ABCA1*, *APOA1*, and *LCAT* was performed using the VarSeq-CNV® caller algorithm. To identify CNVs, the algorithm uses depth-of-coverage information contained within each patient BAM file and compares it to coverage information from a set of “reference” samples that have been previously confirmed to not carry any CNVs. CNVs were called based on comparative increases in read-depth, indicating a duplication of genetic material, and comparative decreases in read-depth, indicating a deletion of genetic material. The criteria in which CNVs were called has been described in detail previously (9).

### Validation of partial gene deletions

#### Breakpoint identification.

To identify the presence of partial gene deletions, primers were designed to flank regions surrounding the putative deletions and were used for PCR amplification (Expand 20 kb^plus^ PCR System; Roche, Mannheim, Germany, catalog no. 11811002001). Forward (F) and reverse (R) primers flanking the deletion junctions were: F1 5′-AGCACGATAGGAAGCATCTTC-3′ and R1 5′-ATCACTGTCTGTGGCAACCAG-3′ (exon 4 deletion); F2 5′- GACCCAGCTTCCAATCTTCATAA-3′ and R2 5′- TAGACAGAATCAGGCCATAATCTG-3′ (exons 8-31 deletion). Gel elec­trophoresis of the PCR products was used as a visual confirmation of the mutant alleles. Sanger sequencing and primer-walking of the PCR products were performed to identify the deletion breakpoints.

#### Sanger confirmation.

Once deletion breakpoints were identified, screening primers spanning the upstream or downstream breakpoint were designed for PCR and Sanger sequencing (supplemental Table S1) to confirm the deletion breakpoint sequences.

### Validation of full-gene deletions

#### Exome sequencing.

Patients with expected full-gene deletions had their DNA samples indexed and pooled using the TruSeq Rapid Exome Kit (Illumina, San Diego, CA; catalog no. 20020616) in preparation for exome sequencing. Sequencing was then performed at the London Regional Genomics Centre (www.lrgc.on.ca; London, ON, Canada), using a NextSeq 500 (Illumina). The same bioinformatic approach described above was used to replicate the CNV call made by the VarSeq-CNV® caller algorithm.

#### Microarray analysis.

Patients with expected full-gene deletions had their DNA samples assessed with the Affymetrix CytoScan^TM^ HD Array (Thermo Fisher Scientific, Waltham, MA) for the genomic region containing the CNV. With >2 million probes on the array, deletions >25 kb can be detected. The microarray was performed following the manufacturer’s instructions at Victoria Hospital (London, ON, Canada), and the resultant data were analyzed using the Chromosome Analysis Suite (ChAS) (version 3.2; Thermo Fisher Scientific). The regions between adjacent probes that differed in copy-number state were marked as containing the approximate breakpoints of the CNV and were used to gauge the approximate size of the deletion.

#### Breakpoint identification.

Once the magnitude of the deletion was established, the approximate location of each breakpoint was estimated. Primers flanking the deletion junction were: F3 5′- CCTGGCTGCTTCTAAGAGCCTATGATC-3′ and R3 5′- TGTCTCTACATGGTCCTCCTTCTGTGC-3′, and were used for PCR amplification (Expand 20 kb^plus^ PCR System, Roche; cat. no. 11811002001). Gel electrophoresis of the PCR products was used as a visual confirmation of the mutant allele. Sanger sequencing and primer-walking of the PCR product were performed to identify the deletion breakpoints.

#### Sanger confirmation.

Once deletion breakpoints were identified, screening primers spanning the upstream or downstream breakpoint were designed for PCR and Sanger sequencing (supplemental Table S1) to confirm the deletion breakpoint sequences.

## RESULTS

### Study subjects

A total of 288 patients with “low HDL cholesterol” or “hypoalphalipoproteinemia” were sequenced with LipidSeq and screened for CNVs disrupting *ABCA1*, *APOA1*, and *LCAT*. Clinical and biochemical characteristics of the four patients identified as carriers for CNVs are shown in **Table 1**.

**TABLE 1.** Clinical and demographic features of subjects with *ABCA1* copy-number variations

	Patient 1	Patient 2	Patient 3	Patient 4
Age	37	34	59	40
Gender	Female	Female	Male	Female
Weight (kg)	56.5	72	—	71.4
Height (cm)	155	156.9	—	172
Waist circumference (cm)	73.7	81.5	—	66
Ethnicity	Northern European, Italian	French-Canadian	French-Canadian	German
Total cholesterol (mmol/L)	8.16	3.30	5.46	4.71
Triglyceride (mmol/L)	2.52	1.01	4.48	5.13
HDL cholesterol (mmol/L)	0.81	0.56	0.47	0.03
LDL cholesterol (mmol/L)	6.20	2.42	3.28	3.54
apo A1 (g/L)	—	0.59	0.60	0.09
apo B (g/L)	—	0.81	1.33	—
Creatine kinase (U/L)	79	—	78	113
Fasting glucose (mmol/L)	4.0	5.4	5.3	4.7
Aspartate transaminase (U/L)	23	20	21	33
Alanine transaminase (U/L)	—	15	15	23
Alkaline phosphatase (U/L)	46	62	—	61
Lp(a) (nmol/L)	—	—	290	363
Co-morbidities	• heterozygous FH (LDLR NM_000527 p.V523M)	• obesity	• hypertension	• TIA at age 37
	• minor carotid intimal thickening		• TIA	• cerebral arteriosclerotic microangiopathy
			• smoking	• hypertension
			• aortic valvular	• juvenile myoclonic epilepsy
			• stenosis	• diffuse nonHodgkin’s lymphoma stage III

Values provided are from first presentation to specialist lipid clinic, or date first obtained. Lp(a) conversions from g/l to nmol/l were done following the conversion factor described by Brown et al. (41). FH, familial hypercholesterolemia; Lp(a), lipoprotein(a); TIA, transient ischemic attack.

### *ABCA1* copy-number variation detection and validation

Analysis of LipidSeq output with the VarSeq-CNV® caller algorithm identified four hypoalphalipoproteinemia patients as carriers of large-scale deletions in *ABCA1* (supplemental Fig. S1). Patient 1 had a heterozygous deletion spanning exon 4; Patient 2 and Patient 3, a pair of siblings, had a heterozygous deletion spanning exons 8 to 31; and Patient 4 had a heterozygous deletion spanning the entire *ABCA1* gene. None of these patients carried rare SNVs in *ABCA1*, *APOA1*, or *LCAT*. There were no CNVs detected in *APOA1* or *LCAT* for any patients in this study.

**Fig. 1.**
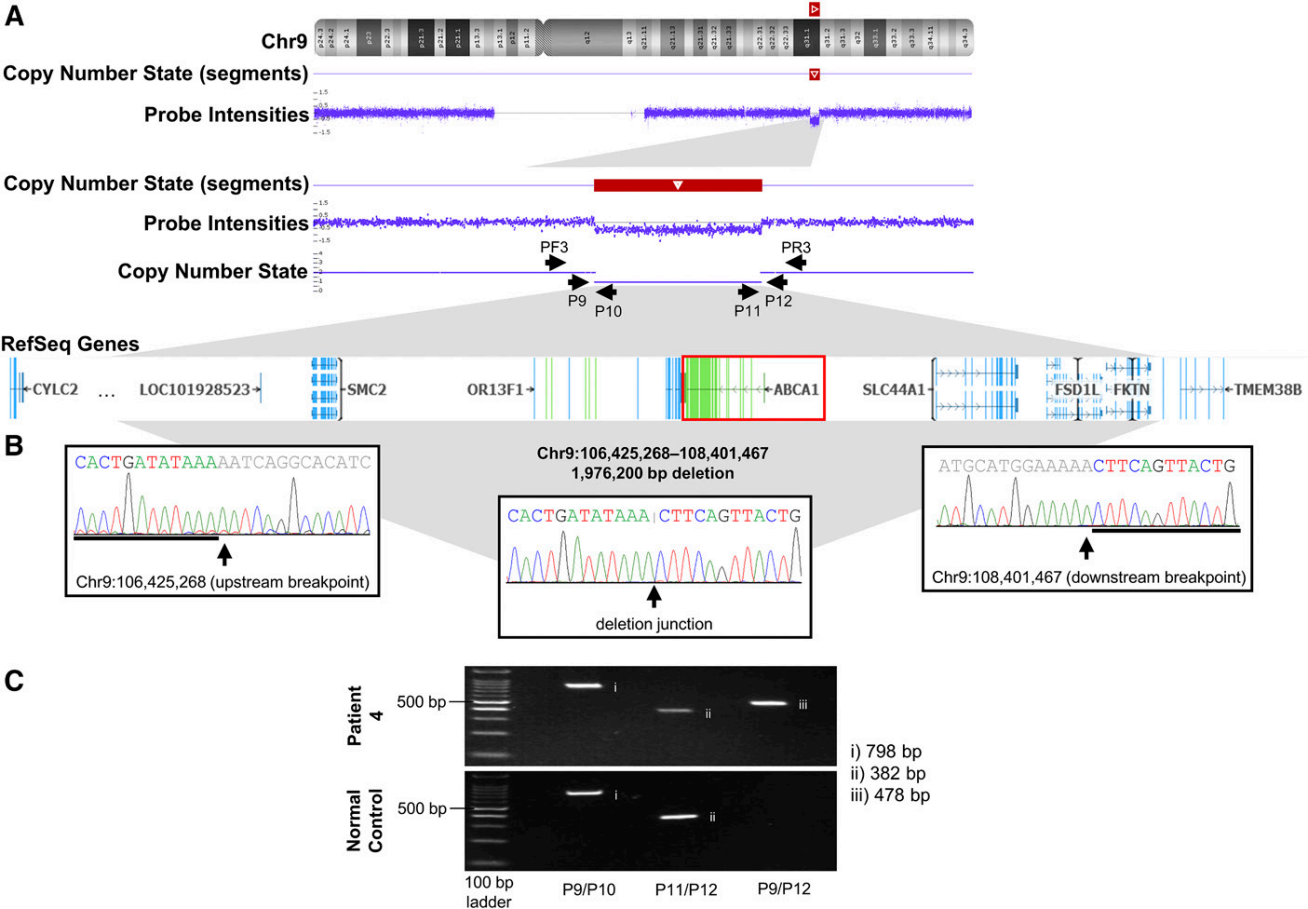
Validation of the full-gene deletion of *ABCA1* in Patient 4 with hypoalphalipoproteinemia. A: Results of the CytoScan^TM^ HD Array, visualized using Chromosome Analysis Suite. Copy Number State (segments) identifies the region containing the CNV. Probe Intensities show a drop in signal, indicating a decrease in copy number at that position, evident under Copy Number State. The black arrows demonstrate the position and orientation of primers used in breakpoint identification and Sanger sequencing. The genes, both coding and noncoding, encompassed by the deletion are evident under RefSeq Genes; the image was taken and modified from Chromosome Analysis Suite and VarSeq®. B: Sanger sequencing results for the forward strand across upstream and downstream breakpoints, and the deletion junction. C: Gel electrophoresis of PCR products across upstream and downstream breakpoints, and the deletion junction. Results from Patient 4 are presented on the top, with results from a normal control on the bottom. Lane 1 contains 100bp ladder, lane 2 contains products across the upstream breakpoint, lane 3 contains products across the downstream breakpoint, and lane 4 contains products across the deletion junction. chr, chromosome; *F*, forward strand; *P*, primer; *R*, reverse strand.

To determine the size of the deletion in Patient 4, the VarSeq-CNV® caller algorithm for exome data was used to confirm the heterozygous absence of *ABCA1* (supplemental Fig. S2), while the CytoScan^TM^ analysis confirmed and replicated the heterozygous nature of this CNV (**Fig. 1A**). Exome sequencing and Cytoscan^TM^ revealed that the CNV was ∼2 Mb in length, and encompassed six additional protein-coding genes, including *SMC2*, *NIPSNAP3A*, *NIPSNAP3B*, *SLC44A1*, *FSD1L*, and *FKTN*.

### Identifying the copy-number variation breakpoints

**Fig. 2.**
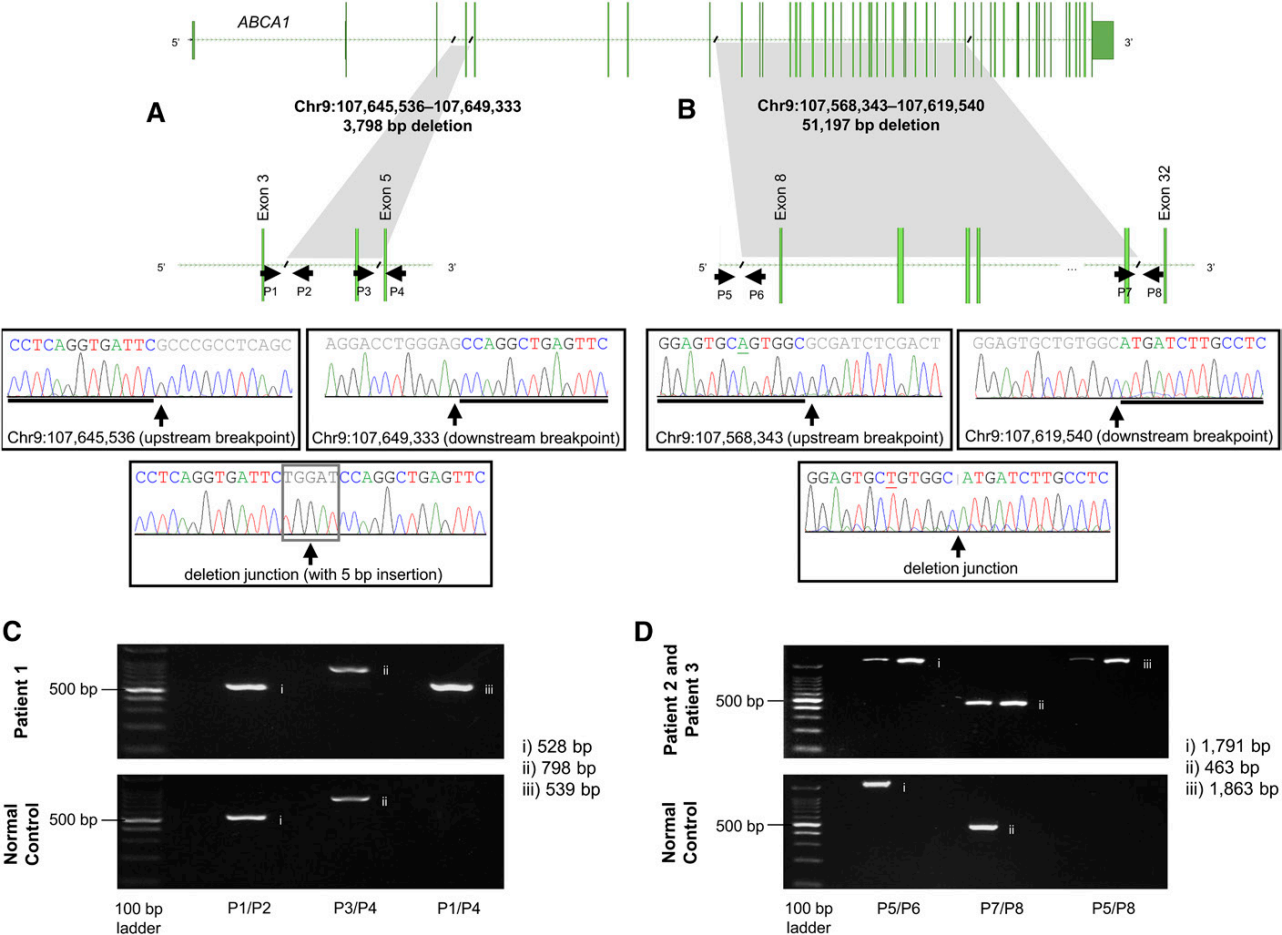
Validation of the partial gene deletions of *ABCA1* in Patients 1, 2, and 3 with hypoalphalipoproteinemia. Sanger sequencing results for the reverse strand across upstream and downstream breakpoints, and the deletion junctions for Patient 1 (A) and Patients 2 and 3 (B). Underlined bases represent polymorphic sites between subjects. The black slashes indicate the sequence breakpoints, while the arrows demonstrate the position and orientation of primers used in breakpoint identification and Sanger sequencing. The gene transcript image was taken and modified from Chromosome Analysis Suite and VarSeq®. Gel electrophoresis of PCR products across upstream and downstream breakpoints, and deletion junctions for Patient 1 (C) and Patients 2 and 3 (D). Results from each patient are presented on the top, with results from a normal control on the bottom. Lane 1 contains 100bp ladder, lane 2 contains products across the upstream breakpoint, lane 3 contains products across the downstream breakpoint, and lane 4 contains products across the deletion junction. chr, chromosome; *P*, primer.

Sanger sequencing across the CNV breakpoints in Patient 1 (**Fig. 2A**), Patients 2 and 3 (Fig. 2B), and Patient 4 (Fig. 1B) revealed the genomic coordinates involved in the deletion event and allowed us to determine the exact size of each CNV (**Table 2**). Screening primers spanning breakpoints were used to distinguish between wild-type and deleted alleles, as indicated in Figs. 1C, 2C, and 2D.

**TABLE 2.** Genomic coordinates and breakpoints of *ABCA1* copy-number variations

CNV	Zygosity state	Breakpoint		
		Genomic coordinates	Length (bp)	HGVS notation
Exon 4	Heterozygous	chr9:107645536 to chr9:107649333	3,798	g.107645536_107649333delinsATCCA
				c.160_301del
				p.Cys54LeufsTer22
Exons 8 to 31	Heterozygous	chr9:107568343 to chr9:107619540	51,197	g.107568343_107619540del
				c.720_4463del
				p.Arg241_Gln1488del
Full deletion	Heterozygous	chr9:106425268 to chr9:108401467	1,976,200	g.106425268_108401467del

The sequences are in the forward-strand orientation, with genomic coordinates based on the hg19 human genome reference build. chr, chromosome; CNV, copy-number variation; HGVS, Human Genome Variation Society.

## DISCUSSION

In 288 patients with hypoalphalipoproteinemia, we identified three rare, large-scale deletions in *ABCA1* in four individuals by applying specialized bioinformatic tools to NGS data. While it is not the first time CNVs have been observed in *ABCA1* (12–26), it is the first report of *ABCA1* CNVs being found specifically in patients with hypoalphalipoproteinemia, and suggests CNVs may be large contributors toward each of these low HDL cholesterol phenotypes.

ABCA1 is a critical player in the reverse cholesterol transport pathway. Found on the surface of macrophages, ABCA1 mediates the transport of free cholesterol out of the cell, where it can be picked up by apo A1, leading to the generation of nascent HDL particles (27). Disruptions to this protein can alter its function and lead to problems with cholesterol efflux and the generation of circulating HDL particles. Rare homozygous variants in this gene have been shown to cause Tangier disease (28–30), while heterozygous mutations can lead to less severe forms of hypoalphalipoproteinemia (28, 31). Given the sizes of our identified CNVs and their predicted consequences on the protein product, they each likely impart a loss of function, leading to a decrease in the generation of HDL particles and an overall decrease in circulating HDL cholesterol.

The smallest CNV deletion is 3,798 bp in size, with its breakpoints in introns 3 and 4, causing a partial loss of both introns, and a full loss of exon 4. The deletion of the coding sequence caused a frameshift and a premature truncation of the protein at the 76th amino acid; 96.7% of the protein is lost. Because our study is limited in that we did not test mRNA levels, protein levels, or protein function, we cannot comment on the exact mechanism by which this *ABCA1* CNV leads to low HDL cholesterol levels; however, given that the CNV produces a premature stop codon, the truncated mRNA could be degraded through the nonsense-mediated decay pathway (32).

The intermediate CNV deletion is 51,197 bp size, with its breakpoints in introns 7 and 31, causing a partial loss of both introns, and a full loss of 23 exons. Because the deletion is in-frame, there is no introduction of a premature stop codon, but 1,248 out of 2,261 amino acids are lost, accounting for 55.2% of the protein. The lost amino acids span from the first extracellular domain to the second, and include the intracellular nucleotide-binding domain, the first regulatory domain, and six transmembrane domains (33). Given the size of the deletion, there are many possibilities for mechanistic dysfunction. One possibility is that apo A1 is unable to interact with ABCA1 through its extracellular domains, while an alternative possibility is that cholesterol cannot be transported out the cell (34–37).

The full-gene CNV deletion is ∼2 Mb and encompasses seven protein-coding genes, including *ABCA1*. In contrast to the previous two CNVs, due to the complete loss of a functional allele, the mechanism of decreased HDL cholesterol may simply be based on a decrease in *ABCA1* expression. As the largest and most severe CNV out of all four patients, it is also interesting to note that the patient carrying this deletion has the most severely decreased levels of HDL cholesterol, at 0.03 mmol/L. However, simple haploinsufficiency seems to be an inadequate explanation for such a severely depressed HDL cholesterol level in this patient. Perhaps the concurrent deletion of several other neighboring genes might help explain the severity of the biochemical phenotype.

When considering the magnitude of each CNV, the size of the genomic deletion correlates to the severity of the HDL phenotype for each patient; however, the corresponding loss of amino acids does not. The patient with the smallest CNV had an HDL cholesterol level of 0.81 mmol/l, while the patients with the intermediate CNV had HDL cholesterol levels of 0.56 mmol/l and 0.47 mmol/l. Additional studies are necessary to fully understand the mechanistic consequences of each CNV, particularly the partial deletions, and how they impact each patients’ HDL phenotype. As well, the severity of each patient’s phenotype may not solely be due to the CNV, but may be influenced by additional genetic or environmental determinants (38). Others have noted a wide range in HDL cholesterol levels, ranging from ∼15 to 70% of normal values among heterozygous carriers of *ABCA1* nonsense mutations resulting in premature protein truncation (39); this inter-individual variation in HDL cholesterol reduction echoes the range of biochemical disturbances seen in the small patient sample studied here. Difficulty in attributing quantitative or pathogenic impact is also encountered in research on heterozygous *ABCA1* SNVs that affect HDL cholesterol; functional studies may help further understanding of the mechanistic impact of an SNV, but even between individuals who share the same genetic variant, there can be substantial differences in HDL cholesterol levels (40). Such differences might result from unmeasured gene X gene interactions, unmeasured gene X environment interactions, or epigenetic, mitochondrial, or microbiome effects.

Our findings implicate a novel form of genetic variation that is likely impacting HDL cholesterol levels, and further emphasizes the complex genetic architecture underlying HDL phenotypes. Understanding that levels of HDL cholesterol can be influenced by rare SNVs, accumulation of common SNPs, and now the presence of rare CNVs, will influence future screening of individuals with extreme HDL phenotypes. Systematic screening for CNVs until recently had heretofore not been feasible due to time-consuming and costly methods (8); improvements to bioinformatic tools have enabled robust analysis of NGS data, leading to comprehensive, simultaneous assessment of multiple types of genetic determinants. These tools will likely reveal further diversity of the genetic basis for other dyslipidemia and metabolic phenotypes. Given their low frequency in our patient cohort, we anticipate that large-scale CNVs, either deletions or insertions, will likely be infrequent among patients with dyslipidemias, but will nonetheless still need to be considered, in addition to small-scale rare genetic variants and polygenic risk.

## Supplementary Material

Supplemental Data
