# Supplementary material for: Large-scale deletions of the ABCA1 gene in patients with hypoalphalipoproteinemia
Source: J Lipid Res. 2018 Jun 4;59(8):1529–35. doi: 10.1194/jlr.P086280 (PMC6071767; doi:10.1194/jlr.P086280)
Supplement: Supplemental Data [file supp_59_8_1529__index.html]

Large-scale deletions of the ABCA1 gene in patients with hypoalphalipoproteinemia — Large-scale deletions of the ABCA1 gene in patients with hypoalphalipoproteinemia — Supplemental Data 

# Large-scale deletions of the *ABCA1* gene in patients with hypoalphalipoproteinemia

## Supplemental Data

- Supplemental Material (.pdf, 903 KB) - Supplemental material to accompany manuscript.
