# Supplementary material for: Large-scale deletions of the ABCA1 gene in patients with hypoalphalipoproteinemia
Source: J Lipid Res. 2018 Jun 4;59(8):1529–35. doi: 10.1194/jlr.P086280 (PMC6071767; doi:10.1194/jlr.P086280)
Supplement: Supplemental Data [file 10.1194_P086280_jlr.P086280-1.pdf]

Supplemental Table S1: Screening primers for *ABCA1* copy-number variations.

CNV	Breakpoint	Primer direction	Primer sequence (5' to 3')	Annealing temperature (°C)	Primer labels in Figure 1 and 2
Exon 4	Upstream	FWD	CCAAATAGCTGAGACTACAGGCATG	60	P1
		REV	GTGATGGTGAAGGTATTTCAG	60	P2
	Downstream	FWD	CATGACTGCATTGGTATAAAGATG	60	P3
		REV	ATCACTGTCTGTGGCAACCAG	60	P4
Exons 8 to 31	Upstream	FWD	GACCCAGCTTCCAATCTTCATAATCCTC	60	P5
		REV	GGTTGCAAAGATCCCTGTAGAG	60	P6
	Downstream	FWD	GAGATATCATGTTGGGAGGGTCTG	60	P7
		REV	GCCACAGTCTGTCCTGTGACTTTAC	60	P8
Full deletion	Upstream	FWD	TATCATGCTACTCAGAACAGCATG	60	P9
		REV	TGGTGATTCTTGTGTGCACAAAG	60	P10
	Downstream	FWD	CAGGATATTACATAGGTAAGCAGG	60	P11
		REV	CTTAATGATAGTGGAAGACAAGGAG	60	P12

The primers listed were designed to flank the two breakpoints for each CNV. The "Breakpoint" listed is relative to the deletion section of the gene. The sequence orientation for P1-P8 are relative to the *ABCA1* gene, while the sequence orientation for P9-P12 are relative to the full chromosome. Abbreviations: CNV = copy-number variation; FWD = forward; REV = reverse.

A

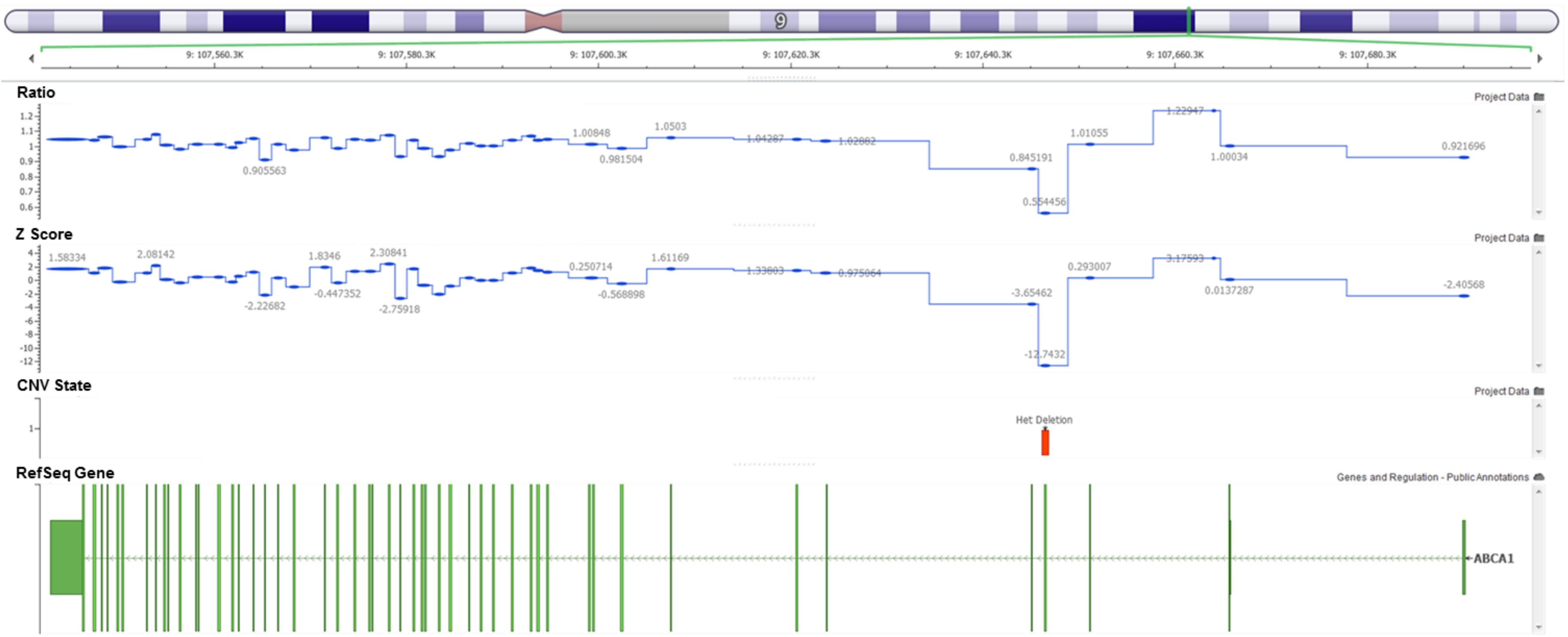

Ratio
Z Score
CNV State
Het Deletion
RefSeq Gene
ABCA1
Project Data
Genes and Regulation - Public Annotations

B

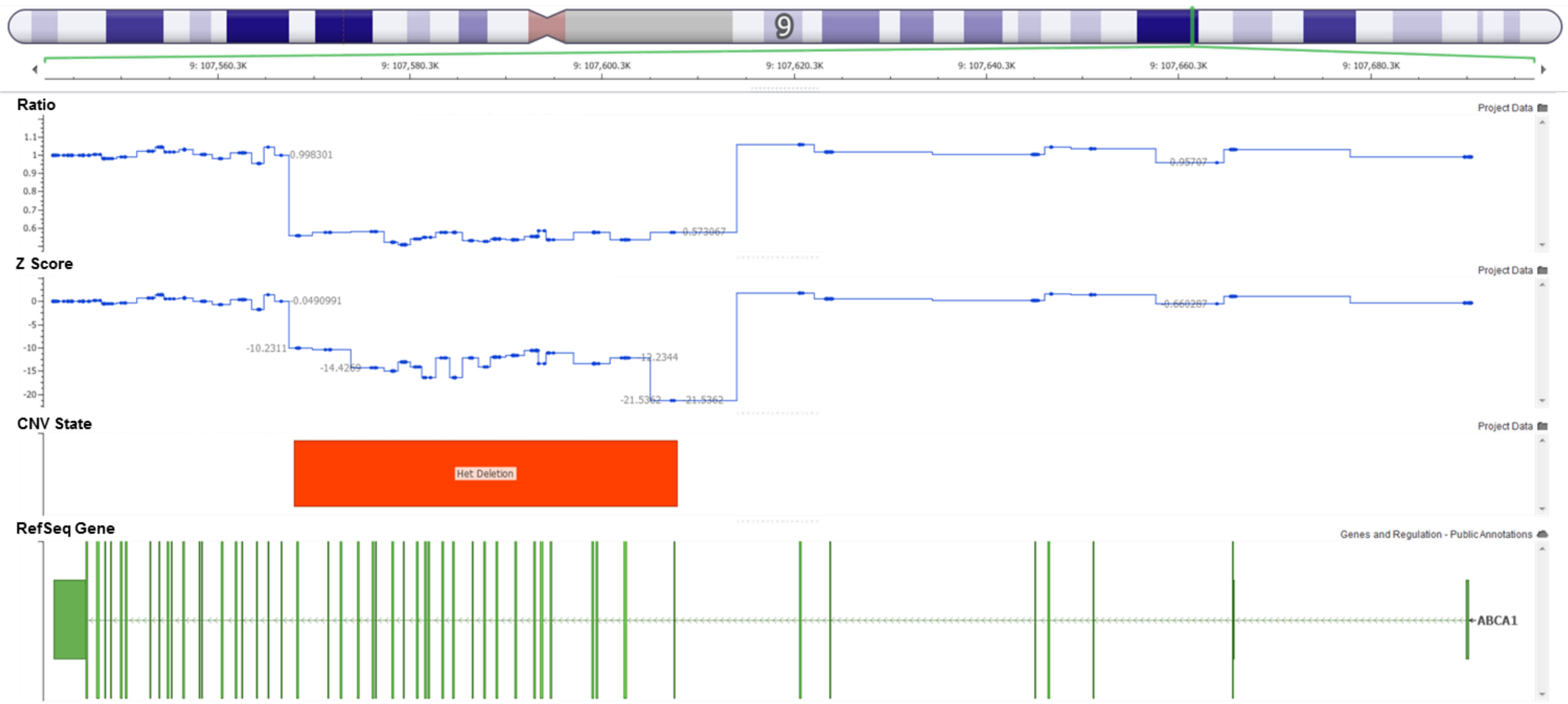
9
9: 107,560.3K
9: 107,580.3K
9: 107,600.3K
9: 107,620.3K
9: 107,640.3K
9: 107,660.3K
9: 107,680.3K
Ratio
Project Data
0.998301
0.573067
0.95707
Z Score
Project Data
-0.0490991
-10.2311
-14.4269
-12.2344
-21.5362
-21.5362
-0.660287
CNV State
Project Data
Het Deletion
RefSeq Gene
Genes and Regulation - Public Annotations
ABCA1

C

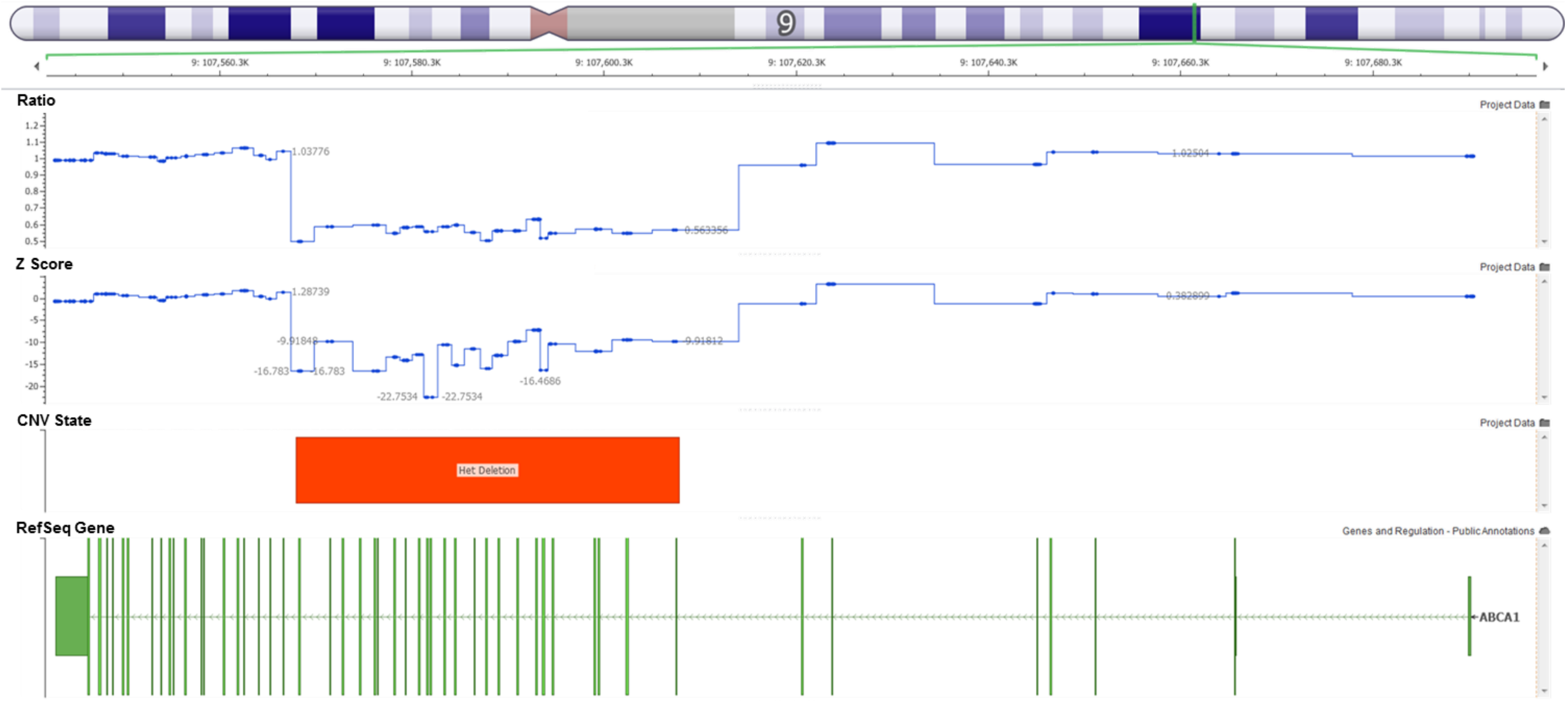
9
9: 107,560.3K
9: 107,580.3K
9: 107,600.3K
9: 107,620.3K
9: 107,640.3K
9: 107,660.3K
9: 107,680.3K
Ratio
Project Data
1.03776
0.563356
1.02504
Z Score
Project Data
1.28739
-9.91848
-16.783
-16.783
-22.7534
-22.7534
-16.4686
-9.91812
0.382899
CNV State
Project Data
Het Deletion
RefSeq Gene
Genes and Regulation - Public Annotations
ABCA1

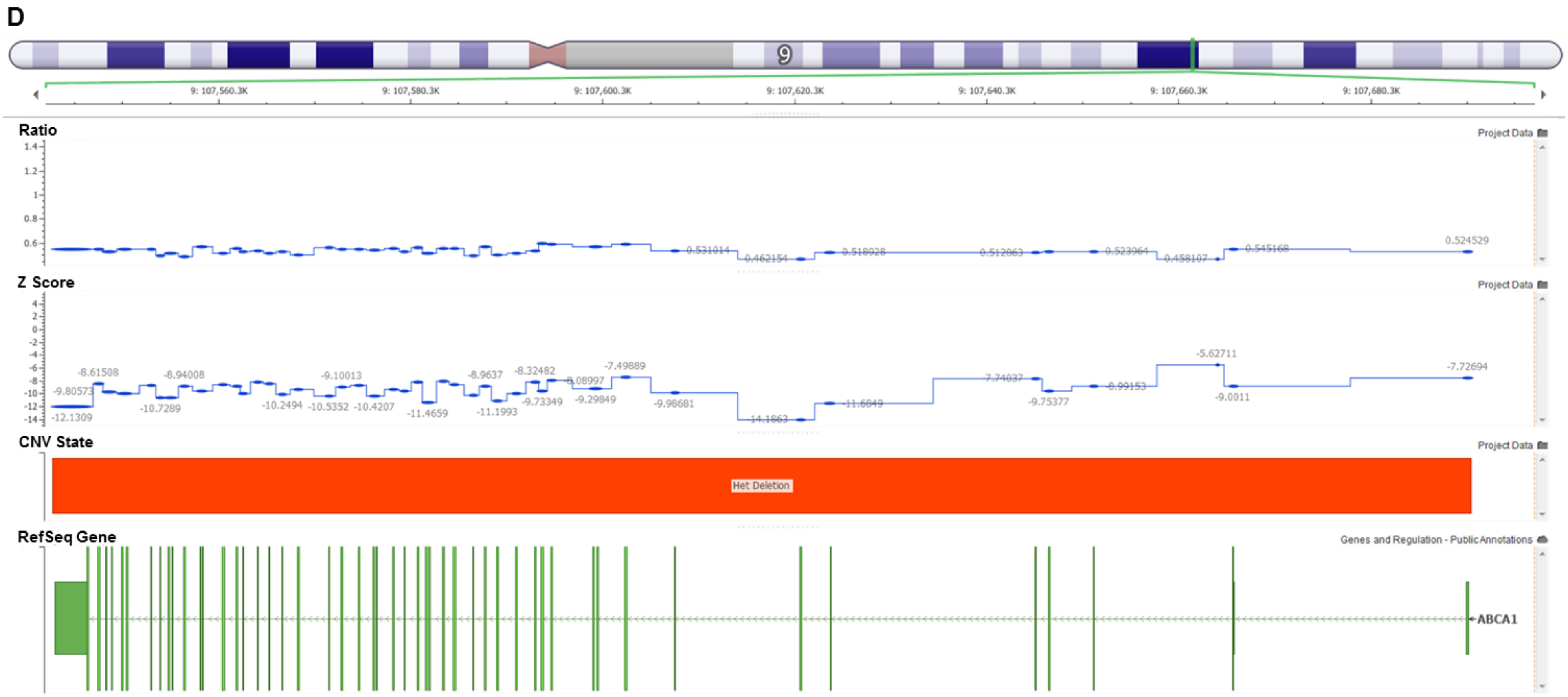

Supplemental Figure S1. Identification of *ABCA1* CNVs using the VarSeq-CNV® caller algorithm on targeted sequencing data. Chr9:107,542,273–107,697,356 (hg19 genome build) is the region visualized in each panel, with the CNV "ratio", "Z score", and "state" available for each subject. **A)** Subject 1, carrier of a heterozygous deletion of exon 4. **B)** Subject 2, carrier of a heterozygous deletion spanning exons 8 to 31. **C)** Subject 3, carrier of a heterozygous deletion spanning exons 8 to 31. **D)** Subject 4, carrier of a heterozygous deletion of the entire *ABCA1* gene. CNV = copy-number variation.

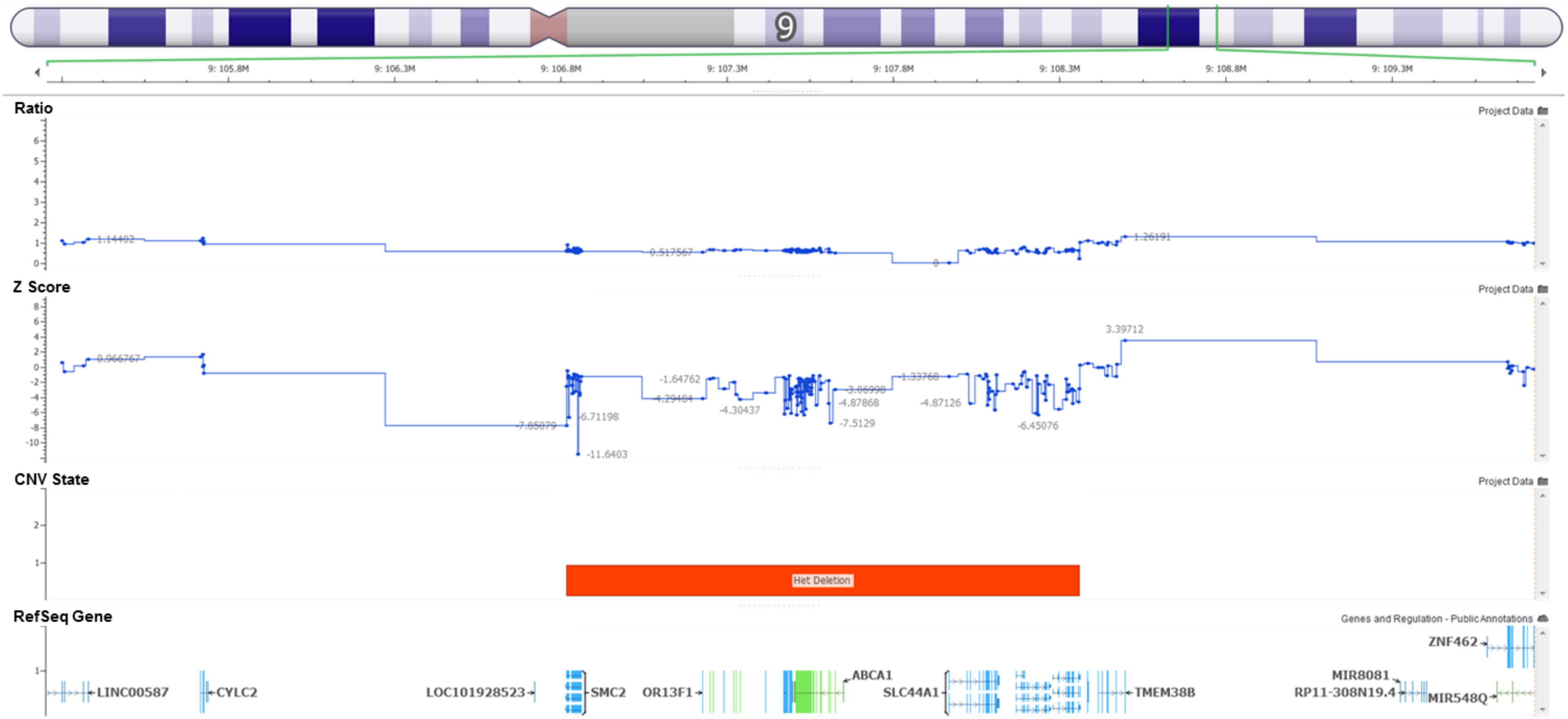

Supplemental Figure S2. Confirmation of the full-gene *ABCA1* CNV using the VarSeq-CNV® caller algorithm on exome data. Chr9:105,295,869–109,769,141 (hg19 genome build) is the region visualized, with the CNV “ratio”, “Z score”, and “state” available for the subject. Subject 4, carrier of a heterozygous deletion of the entire *ABCA1* gene and surrounding loci. CNV = copy-number variation.
